# Role of Households with Children in Community Spread of Multidrug-Resistant Enterobacterales, St. Louis, Missouri, USA

**DOI:** 10.3201/eid3206.251655

**Published:** 2026-06

**Authors:** Barrett Breeze, Ahmed Babiker, Sreenivas Konda, Alaina L. Robinson, Stefan J. Green, Catherine C. Babbs, Federico Cunha, Katherine Y. Shen, India Shepherd Hammond, Stephanie A. Fritz, Latania K. Logan

**Affiliations:** Emory University School of Medicine, Atlanta, Georgia, USA (B. Breeze, C.C. Babbs, F. Cunha, K.Y. Shen, I.S. Hammond, L.K. Logan); Rush University Medical Center, Chicago, Illinois, USA (A. Babiker, S.J. Green); The University of Chicago, Chicago (S. Konda); Washington University School of Medicine, St. Louis, Missouri, USA (A.L. Robinson, S.A. Fritz); Children’s Healthcare of Atlanta, Atlanta, Georgia, USA (L.K. Logan)

**Keywords:** Enterobacterales, community, beta-lactamases, children, antibiotic resistance, antimicrobial resistance, epidemiology, bacteria, bacterial infections, United States

## Abstract

Community-acquired multidrug-resistant (MDR) Enterobacterales bacteria are an increasing public health concern, yet whether households play a role in community spread remains unclear. We investigated 150 households with children in St. Louis, Missouri, USA, for MDR Enterobacterales. We cultured swab specimens from household members and environmental surfaces for identification and antimicrobial susceptibility testing. We also performed whole-genome sequencing in the 53 (35%) households where >1 MDR Enterobacterales species were recovered. *Enterobacter hormaechei* predominated, followed by *Klebsiella pneumoniae* and *Pantoea* species. Whole-genome sequencing revealed closely related strains shared between persons and environmental surfaces, suggesting potential intra-household transmission. We identified >1 horizontal gene transfer event between Enterobacterales genera within a household. On multivariable analysis, households that had children attending daycare, a member with an ADHD diagnosis, and dog ownership were associated with increased odds of household MDR Enterobacterales colonization. Households likely serve as major contributors in acquisition and community spread of MDR Enterobacterales.

Multidrug resistant (MDR) Enterobacterales bacteria have emerged as a serious public health threat. Of major concern is the increasing incidence of extended-spectrum cephalosporin resistance (ESCR) in Enterobacterales. This resistance pattern is commonly associated with the production of extended-spectrum β-lactamases (ESBL), antimicrobial resistance genes (ARGs) carried on mobile genetic elements (MGEs) such as plasmids, which often carry other ARGs. The incidence of infections has increased dramatically during the past decade, reaching nearly 200,000 infections and >9,000 deaths per year in the United States (*1*). Although ESBL Enterobacterales were once largely healthcare-associated pathogens, during the past 2 decades, most ESBL Enterobacterales infections have been caused by clinically and genetically distinct strains that have emerged in the community (*2*).

Adding to the complexity of this group of pathogens, the determinants of resistance yielding the ESCR phenotype might be chromosomal, transferable ARGs on MGEs, or contain both, and are often MDR. Invasive infections caused by community- acquired ESCR Enterobacterales strains are increasingly being reported in young children and persons without major healthcare exposures (*3*,*4*). Of note, those strains are resistant to antimicrobial drugs that are uncommonly used in children; therefore, overuse is unlikely to be driving this resistance.

Factors promoting MDR and ESCR Enterobacterales acquisition and infection in the community are largely unknown. However, in households with an adult known to have acquired ESBL Enterobacterales from healthcare exposures, transmission incidence has been described as upwards of 67% (*5*,*6*). Furthermore, in a multicenter investigation of MDR (primarily ESBL) Enterobacterales in children from Chicago, Illinois, USA, we found that MDR Enterobacterales acquisition reflected geographic clustering and lacked association with the factors driving primary acquisition in adults (e.g., antimicrobial drug and healthcare exposures) (*7*,*8*). We also found that, compared with antimicrobial-sensitive Enterobacterales infections in children, *bla*_CTX-M-9_-type ESBL Enterobacterales infections were nearly 5 times more likely to be community-acquired (*8*). To devise strategies to prevent community-acquired MDR Enterobacterales infections, we must first understand key reservoirs for acquisition, including sources of transmission outside of healthcare settings, the role of the natural and built environment in pathogen transmission, and epidemiologic factors associated with community-acquired MDR Enterobacterales acquisition, transmission, and infection.

We believe households are major drivers of MDR Enterobacterales spread in the community and that environmental surfaces are major reservoirs of MDR Enterobacterales in households. That hypothesis is supported by observations that MDR Enterobacterales strains can persist on surfaces in healthcare environments for up to 30 months, likely contributing to healthcare-associated transmission (*9*–*11*). Relevant to those findings, prior studies of household transmission of community-acquired methicillin-resistant *Staphylococcus aureus* (MRSA) in St. Louis, Missouri, USA, have demonstrated that multiple environmental surfaces serve as reservoirs for community-acquired MRSA transmission (*12*–*15*). In addition, households with a higher burden of environmental community-acquired MRSA contamination were 4-fold more likely to enable transmission (*15*). In addition, we believe that critical epidemiologic risk factors for colonization, including the presence of preschool-age children who attend daycare (*16*) and having a pet dog in the household, would be associated with MDR Enterobacterales colonization (*17*).

We used a biorepository of human and household surface samples and associated epidemiologic metadata collected from households of pediatric and adult community participants to understand the clinical and molecular epidemiology of community-acquired MDR Enterobacterales, with a focus on ESCR Enterobacterales. We highlight the household whole-genome sequencing (WGS) data for *Enterobacter* spp., a well-known cause of healthcare-associated infections, which might represent an underrecognized source of community acquisition of antimicrobial drug resistance.

## Methods

### Study Settings and Population

We used a biorepository and detailed epidemiologic metadata from a well-curated population of 150 otherwise healthy children with community-acquired *S. aureus* infections and their household contacts (n = 489) enrolled in the SHINE study from 2015–2021 in metropolitan St. Louis, Missouri, USA (Clinicaltrials.gov, identification no. NCT02572791). Through the SHINE study, research visits were conducted in participants’ homes. Children were defined as 0–18.99 years old. Detailed clinical and epidemiologic data were collected from each participant, including demographics, medical history, topical and systemic antimicrobial drug use, activities outside of the home, personal hygiene practices, and interactions between household contacts. Detailed information regarding household characteristics were also collected, including household environmental cleaning practices, renting versus owning, number of bedrooms, and the presence and characteristics of pets. All data were collected prospectively through the SHINE study and were entered into REDCap, a secure, HIPAA-compliant, web-based data application (*18*). At each study visit, colonization samples were collected from the anterior nares, axillae, and inguinal folds by using BD Eswabs (BD, https://www.bd.com). Rectal or perirectal swab specimens were not collected.

Through the SHINE study, samples were collected from up to 21 household environmental surfaces. A 250-µL aliquot of liquid amies transport media from each swab was enriched and suspended in tryptic soy broth plus 20% glycerol and frozen at −80°C. For this study, we used samples from households collected at SHINE study enrollment (natural history phase). The Washington University Institutional Review Board approved study procedures. Informed consent was obtained for all participating household members.

### Bacterial Isolates and Antimicrobial Susceptibility Testing

We chose to use inguinal fold swab specimens on the basis of a higher likelihood of colonization because of proximity to the rectum. We transferred swab suspension samples and environmental surface samples to tryptic soy broth and incubated them overnight at 37°C. We used a 1-μL inoculation loop to streak the enriched broth onto membrane fecal coliform agar to evaluate the presence of Enterobacterales bacteria. In addition, we spread-plated 100 μL of the broth onto CHROMagar ESBL media (CHROMagar, https://www.chromagar.com) (*19*). We incubated plates at 37°C for 16–20 hours, subcultured the resulting colonies on CHROMagar, and incubated them overnight at 37°C. After incubation, we resuspended the colonies in saline and adjusted to a 0.5–0.63 McFarland standard for isolate identification and antimicrobial susceptibility testing using the automated VITEK 2 system (bioMérieux, https://www.biomerieux.com), according to the manufacturer’s instructions.

We used a broad definition for MDR Enterobacterales because isolates were ESCR, with some demonstrating resistance to carbapenems, all of which were resistant to >2 antimicrobial classes. We selected the colonies we defined as MDR Enterobacterales for further testing (*20*). We chose a subset of the resistant isolates for WGS if they were from households where Enterobacterales were recovered from >1 surface or person. We extracted DNA by using the QIAGEN DNeasy Blood and Tissue Kit (QIAGEN, https://www.qiagen.com) according to the manufacturer’s instructions and shipped the extracted genomic DNA to the Rush University Medical Center Genomics and Microbiome core facility (Chicago, Illinois, USA) for WGS.

### WGS

We performed bacterial WGS by using standard shotgun sequencing methods, as described previously (*19*). In brief, we prepared genomic DNA for sequencing by using a NEXTFLEX rapid XP DNA sequencing kit (Revvity, https://www.revvity.com) implemented on a Sciclone G3 NGSx iQ (Revvity) workstation. We normalized DNA inputs to 10 ng and used 10 cycles of amplification. After magnetic bead cleanup (0.8× ratio of beads to template, vol:vol), we sequenced libraries by using an Illumina NovaSeq X (Illumina, https://www.illumina.com) instrument using a 10 billion cluster flowcell lane. Libraries were created by the Rush University Medical Center for Genomics and Microbiome core facility, and sequencing was performed by the DNA Services Core, Carver Biotechnology Center, University of Illinois Urbana-Champaign (Urbana-Champaign, Illinois, USA).

### Bioinformatic Analysis

FASTQ files underwent QC screening, and we assembled and analyzed them by using the Bactopia pipeline (*21*). In brief, we only further analyzed FASTQ files if they met the following parameters: estimated genome coverage >20×, mean per-read quality score >Q12, mean post-trimming read length >49 bp, and <500 total contigs. We quality filtered Illumina reads by using Trimmomatic (*22*) and assembled de novo by using SPAdes (*23*). We used Prodigal (*24*) to predict gene sequences and annotated them with Prokka (*25*). We assessed antimicrobial resistance content by using AMRFinder Plus (*26*). We defined core genes by using Roary (*27*). We generated a phylogenetic tree on the basis of a core gene alignment by using IQtree (*28*). We generated a maximum-likelihood tree by running 1,000 bootstrap replicates under the generalized time-reversible model of evolution. We inferred the maximum-likelihood phylogeny from the core genome alignment by using IQ-TREE under the Hasegawa–Kishino–Yano nucleotide substitution model with 1,000 ultrafast bootstrap replicates and 1,000 SH-like approximate likelihood ratio test replicates. We visualized and annotated the tree by using iTOL version 4 (*29*). We calculated the core genome pairwise single-nucleotide polymorphism distance for each sample with snp-dists (*30*) and completed pangenome wide comparison of genomes by using Scoary (*31*). We reconstructed, typed, and clustered plasmids by using MOB-suite (*32*). We performed clustering for plasmids with a mash distance <0.05 with >85% similarity in length. We considered plasmid transfer if a mobilizable or conjugative plasmid within the same primary cluster was detected in a different species within the same household. Sequence data are available in the National Center for Biotechnology Information Sequence Read Archive (https://www.ncbi.nlm.nih.gov/sra; BioProject no. PRJNA1257399).

### Statistical Analysis

We used a retrospective case–control study design to assess factors associated with household colonization with MDR Enterobacterales. We presented the descriptives numerical variable by mean +SD and of categorical variables by counts and percentages (*33*). We conducted bivariate analysis by using a 2-sample t-test and χ^2^ test for independence. We conducted multivariate analysis of the binary outcome by using logistic regression. We selected covariates in the final logistic regression by the LASSO method (*34*,*35*) first, and then by clinical importance of the variables because of a large number of covariates associated with households and household members and pets (*12*,*36*).

We performed logistic regression analysis to identify significant household clinical and nonclinical factors associated with household colonization with MDR Enterobacterales. On the basis of the observation numbers, robustness to reporting, prevarication bias, and biologic plausibility regarding Enterobacterales carriage, we selected a covariate with a p<0.25 in univariate analysis for inclusion in a multivariable model by using a manual forward selection approach in which variables having a p = 0.10 remained in the model. We addressed potential confounding effects by retaining variables whose exclusion from the models changed the effect of the other covariates by >10%. We tested interactions between independent variables and expanded the final multivariable models to include the significant (p<0.10) interaction terms. We checked for any collinearities between independent variables before multivariable analysis and made selections between collinear variables on the basis of an improved model fit as shown by the Akaike information criterion and Bayesian information criterion (*36*,*37*). We expressed bivariate and multivariate associations as odd ratios (ORs) and corresponding 95% CIs. Statistical significance was indicated by p<0.05.

## Results

### Characteristics of Households in the Study Population

We analyzed 150 households and their characteristics. The mean age of the 639 participants was 20.86 (SD +6.30); for race, 73% identified as White, 24% as Black, and 3% as mixed race or Asian descent. In addition, >1 household member held a college degree (43%), private insurance (77%) or Medicaid insurance (31%). All households had >1 child (52% of all participants were children), 71% had >1 child in daycare, 51% had >1 dog, and the average number of residents per household was 4.31 (SD +1.34).

We univariately analyzed ≈100 variables from clinical and epidemiologic data. Of those, we summarized 20 risk factors from the univariate analysis results along with their bivariate associations on the basis of the presence and absence of MDR Enterobacterales (Table 1).

**Table 1:** Descriptive statistics by HHs with (cases) and without (controls) MDR Enterobacterales in study of role of HHs with children in community spread of multidrug-resistant Enterobacterales, St. Louis, Missouri, USA*

Variable	Total HHs, n = 150	Controls, n = 97	Cases, n = 53	Odds ratio (95% CI)	p value
Mean HH size (SD)	4.31 (1.34)	4.29 (1.26)	4.36 (1.48)	1.04 (0.81–1.33)	0.76
HH members’ mean age, years (SD)	20.86 (6.30)	21.3(5.97)	19.95(6.82)	0.96 (0.91–1.02)	0.19
HH mean home size in square feet (SD)	1,785 (1,021)	1,953(1,027)	1,481 (946)	1.02 (1.01–1.03)	0.01
HH mean number of rooms (SD)	9.85 (3.35)	10.32 (3.58)	9.00 (2.72)	0.88 (0.78–0.98)	0.02
HH mean square feet per person (SD)	441 (262)	478 (259)	374 (255)	0.98 (0.97–0.99)	0.02
HH size >5 membership					
No	130 (87)	87 (90)	43 (81)		
Yes	20 (13)	10 (10)	10 (19)	2.02 (0.77–5.29)	0.15
HH homeownership					
No	56 (37)	30 (31)	26 (49)		
Yes	94 (63)	67 (69)	27 (51)	0.46 (0.23–0.92)	0.03
HHs with >1 health conditions					
No	24 (16)	14 (14)	10 (19)		
Yes	126 (84)	83 (86)	43 (81)	0.73 (0.30–1.81)	0.48
HHs with >1 ADHD member					
No	94 (75)	66 (80)	28 (65)		
Yes	32 (25)	17 (20)	15 (35)	2.08 (0.91–4.76)	0.08
HHs with >1 antimicrobial prescription within 12 mo					
No	85 (57)	49 (51)	36 (68)		
Yes	65 (43)	48 (49)	17 (32)	0.48 (0.24–0.96)	0.04
HHs with >1 emergency room visit within 12 mo					
No	10 (6.7)	7 (7.2)	3 (5.7)		
Yes	140 (93)	90 (93)	50 (94)	1.3 (0.34–6.22)	0.72
White households					
No	40 (27)	17 (18)	23 (43)		
Yes	110 (73)	80 (82)	30 (57)	0.28 (0.13–0.58)	<0.01
HHs with >1 college degree					
No	86 (57)	53 (55)	33 (62)		
Yes	64 (43)	44 (45)	20 (38)	0.73 (0.36–1.44)	0.37
HH with >1 professional degree					
No	102 (68)	62 (64)	40 (75)		
Yes	48 (32)	35 (36)	13 (25)	0.58 (0.26–1.20)	0.15
HHs with >1 private insurance member					
No	35 (23)	16 (16)	19 (36)		
Yes	115 (77)	81 (84)	34 (64)	0.35 (0.16–0.77)	0.01
HHs with >1 Medicaid recipient					
No	104 (69)	75 (77)	29 (55)		
Yes	46 (31)	22 (23)	24 (45)	2.82 (1.38–5.85)	0.01
HHs with >1 child attending daycare					
No	44 (29)	30 (31)	14 (26)		
Yes	106 (71)	67 (69)	39 (74)	1.25 (0.60–2.69)	0.56
HHs with >1 dog					
No	74 (49)	51 (53)	23 (43)		
Yes	76 (51)	46 (47)	30 (57)	1.45 (0.74–2.86)	0.28
HHs with >1 cat					
No	125 (83)	78 (80)	47 (89)		
Yes	25 (17)	19 (20)	6 (11)	0.52 (0.18–1.34)	0.2

*Values are no. (%) except as indicated. HH, household; MDR, multidrug resistant.

### Characteristics of Bacterial Isolates at the Household Level

We tested 3,201 samples from 627 humans and 2,574 surfaces in 150 households. Enterobacterales strains phenotypically identified as MDR had been recovered from 53 (35%) of 150 households. Of the 120 MDR Enterobacterales isolated from 53 households, most were *Enterobacter* spp. (71%, n = 85), *Pantoea* spp. (12%, n = 14), and *Klebsiella* spp. (8%, n = 10). The household surfaces most commonly harboring MDR Enterobacterales were the kitchen sink faucet handle (20.7%), sofa (9%), bedsheets (6%), oven door handle (6%), and refrigerator door handle (3%). MDR Enterobacterales were identified in the inguinal folds of 25 (4%) household members.

### WGS Analysis of MDR Enterobacterales strains

We chose a subset of isolates for WGS if they were recovered from households where MDR Enterobacterales were found on >1 household surface or household member. Of the 94 samples sequenced, 93 passed pipeline QC metrics. Of those, 76 were Enterobacterales on the basis of genome taxonomy database toolkit taxonomic classification (Table 2). The most common species detected were members of the *Enterobacter cloacae* complex (most were *E. hormaechei* [N = 47]), followed by *K*. *pneumoniae* (N = 10). All isolates were ESCR; 10 were also resistant to >1 carbapenem, none of which were found to contain a transmissible carbapenemase gene.

**Table 2:** Enterobacterales species detected within households by whole-genome sequence analysis in study of role of households with children in community spread of multidrug-resistant Enterobacterales, St. Louis, Missouri, USA

Species	No. (%), n = 76
*Enterobacter hormaechei*	47 (61.8)
*Enterobacter mori*	1 (1.3)
*Enterobacter quasihormaechei*	1 (1.3)
*Enterobacter roggenkampii*	1 (1.3)
*Klebsiella pneumoniae*	10 (13.2)
*Klebsiella variicola*	1 (1.3)
*Serratia marcescens*	4 (5.3)
*Serratia bockelmannii*	3 (3.9)
*Pantoea septica*	3 (3.9)
*Pantoea dispersa*	1 (1.3)
*Pantoea piersonii*	1 (1.3)
*Proteus mirabilis*	2 (2.6)
*Citrobacter braakii*	1 (1.3)

We conducted a relatedness analysis for all species or sequence types (STs) with >4 isolates (*K. pneumoniae* [n = 10], *S. marcescens* [n = 4], *E. hormaechei* ST50 [n = 5] and ST108 [n = 5]). This analysis revealed clustering of isolates within the same households from multiple surfaces and household members (Appendix Table 1).

We reconstructed, typed, and clustered plasmid sequences to assess whether plasmids with similar identity were found across different species isolated from the same household. A total of 251 plasmids were reconstructed across 69 genomes into 100 unique clusters. Across those clusters, we detected >1 potential plasmid transfer event. A plasmid with the same conjugative relaxase type and an identical mash distance to the nearest MOB-suite database reference (GenBank accession no. CP032172) was detected in a *K. pneumoniae* isolate recovered from the inguinal fold of a participant child and *Proteus mirabilis* isolate recovered from the inguinal fold of the child’s mother.

Further analysis of the recovered *E. hormaechei* strains revealed a diversity of STs, with 23 unique STs detected (Figure; Appendix Table 2, Figure). Only 2 major STs (defined as >5 isolate per ST) were detected: ST50 (n = 5 isolates) and ST108 (n = 5 isolates). The remaining 47 were distributed across minor STs (n < 5 isolates per ST). All isolates had a *bla*_ACT_ variant *ampC* gene detected. Among the 47 isolates, 15 were detected from a human source (3 from inguinal folds, 12 from bedsheets) and 32 from an environmental source. ST distribution, AMR gene count, and plasmid count was similar among environmental and human isolates (Appendix Table 2). To assess differential gene content between environmental and human isolates, we conducted a pangenome analysis. However, no candidate genes remained statistically significant after Benjamini-Hochberg correction (false discovery rate <0.05). *K*. *pneumoniae*, the second largest group of strains assessed by WGS revealed primarily *bla*_SHV-ESBL_ and most were also MDR (Table 3).

**Figure:**
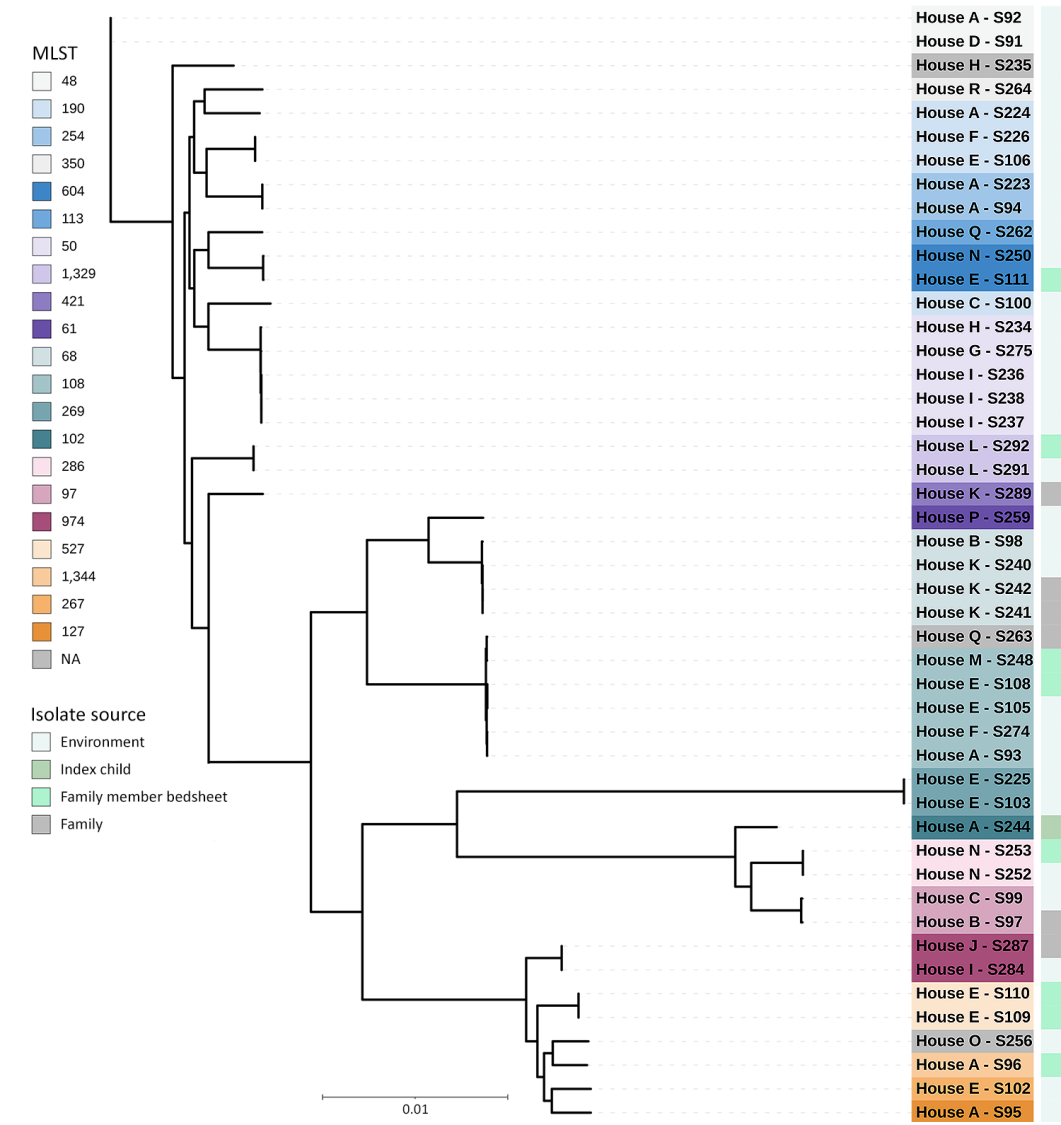
Phylogenetic tree based on core gene alignment for isolated *Enterobacter hormaechei* species (n = 47) in study of the role of households with children in community spread of multidrug-resistant Enterobacteriaceae, St. Louis, Missouri, USA. Scale bar represents nucleotide substitutions per site. MLST, multilocus sequence type; NA, not available.

**Table 3:** *Klebsiella pneumoniae* characteristics by isolate source in study of role of households with children in community spread of multidrug-resistant Enterobacterales, St. Louis, Missouri, USA*

Characteristic	Total, n = 10	Environmental source, n = 7	Human source, n = 3
MLST			
Unknown	2 (20)	2 (29)	0
1380	1 (10)	1 (14)	0
20	1 (10)	1 (14)	0
29	2 (20)	1 (14)	1 (33)
461	1 (10)	1 (14)	0
466	1 (10)	1 (14)	0
678	2 (20)	0	2 (67)
Median AMR gene count per isolate (IQR)	5 (4.25–5)	5 (3.5–5)	5 (5–5.5)
AMR resistance determinants			
β-lactam AMR determinants	10 (100)	32 (100)	15 (100)
*bla*_SHV_, unspecified	2 (20)	1 (14)	1 (33)
*bla*_SHV-1_	1 (10)	1 (14)	0
*bla*_SHV-187_	1 (10)	1 (14)	0
*bla*_SHV-26_	1 (10)	1 (14)	0
*bla*_SHV-33_	1 (10)	1 (14)	0
*bla*_SHV-36_	2 (20)	2 (29)	0
*bla*_SHV-41_	2 (20)	0	2 (67)
*bla*_TEM-10_	2 (20)	1 (14)	1 (33)
Fosfomycin AMR determinants	10 (100)	7 (100)	3 (100)
* fosA*	6 (60)	3 (43)	3 (100)
* fosA10*	4 (40)	4 (57)	0
Multiclass AMR determinants			
* emrD*	10 (100)	7 (100)	3 (100)
Phenicol/quinolone AMR determinants	7 (70)	4 (57)	3 (100)
* oqxA*	3 (30)	2 (29)	1 (33)
* oqxA3*	1 (10)	1 (14)	0
* oqxA10*	3 (30)	1 (14)	2 (67)
* oqxB*	2 (20)	0	2 (67)
* oqxB19*	2 (20)	2 (29)	0
* oqxB25*	3 (30)	2 (29)	1 (33)
Trimethoprim AMR determinants	1 (10)	1 (14)	0
* dfrA50*	1 (10)	1 (14)	0
Median plasmid count per isolate (IQR)	3.5 (3–4.75)	4 (3.5–5)	3 (2–3)
Plasmids			
Col440I	5 (50)	5 (71)	0
Col440II	3 (30)	3 (43)	0
IncFIB(K)	10 (100)	7 (100)	3 (100)
IncFIB(Mar)	1 (10)	1 (14)	0
IncFIB(pKPHS1)	1 (10)	1 (14)	0
IncFIB(pQil)	1 (10)	1 (14)	0
IncFII	3 (30)	1 (14)	2 (67)
IncFII(K)	8 (80)	6 (86)	2 (67)
IncFII(Yp)	1 (10)	1 (14)	0
IncHI1B	1 (10)	1 (14)	0
IncR	2 (20)	2 (29)	0
IncX5	1 (10)	1 (14)	0
repA	2 (20)	1 (14)	0

*Values are no. (%) except as indicated. AMR, antimicrobial resistance; IQR, interquartile range; MLST, multilocus sequence type.

### Analysis of Factors Associated with MDR Enterobacterales

The 53 households with MDR Enterobacterales (cases) were compared to 97 households without MDR Enterobacterales (controls). Although 94 of 150 households identified having >1 family member with >1 health conditions (Table 1; Appendix Table 3), none of those conditions were found to be associated with MDR Enterobacterales colonization on bivariate analysis; primary conditions reported were often mild or common such as asthma and seasonal allergies. However, households reporting >1 antimicrobial drug prescription in the previous 12 months were more common in controls than cases and was inversely associated with MDR Enterobacterales colonization (OR = 0.48, 95% CI 0.24–0.96; p = 0.04). Having smaller homes, fewer rooms, and lower square feet per person were positively associated with MDR Enterobacterales colonization on bivariate analysis.

In the final multivariable logistic regression model (Table 4), factors found to be associated with a lower likelihood of MDR Enterobacterales colonization were households identifying as predominantly White race (adjusted OR [aOR] = 0.18, 95% CI 0.06–0.49; p<0.01) and having ≥1 member of the family with private insurance trended toward significance and was included in the final model (aOR = 0.44, 95% CI 0.16–1.22; p = 0.11). Factors associated with increased risk for household colonization with MDR Enterobacterales included having >1 with a diagnosis of ADHD (aOR = 3.47, 95% CI 1.34–9.41; p = 0.01), >1 minor attending daycare (aOR = 2.86, 95% CI 1.07–8.38, p = 0.04), and >1 dog (aOR = 3.31, 95% CI 1.27–3.31; p = 0.02). Similar differences can be found between aOR and OR (Appendix Table 4).

**Table 4:** Multivariable logistic regression model for households with resistant Enterobacterales in study of role of households with children in community spread of multidrug-resistant Enterobacterales, St. Louis, Missouri, USA

Variable	Odds ratio (95% CI)	p value
White households	0.18 (0.06–0.49)	<0.01
Households with private insurance	0.44 (0.16–1.22)	0.11
Households with >1 ADHD member	3.47 (1.34–9.41)	0.01
Household with >1 minors and attending daycare	2.86 (1.07–8.38)	0.04
Households with >1 dogs	3.31 (1.27–9.41)	0.02

## Discussion

In this study, we focused on understanding the role of the household in acquisition and transmission of MDR Enterobacterales, factors associated with increased or decreased risk for household colonization, the principal genetic determinants, and the relatedness of MDR Enterobacterales strains in community settings. Our research program investigates antimicrobial drug resistance in the community through a One Health lens (*38*).

Enterobacterales that exhibit higher level resistance are designated as high priority in the 2024 WHO priority report (*39*). Of note, studying colonizing isolates overcomes biases that are intrinsic to surveillance systems reliant on passively collected clinical isolates (*40*), which is critical because colonization frequently precedes infection, and asymptomatic carriers can serve as sources of onward MDR organism transmission. In particular, colonization with MDR and ESCR Enterobacterales in children can last months or years, and silent dissemination of transmissible ARGs in Enterobacterales has been described in healthy pediatric populations (*41*–*43*).

We found that, in households not known to previously harbor MDR Enterobacterales, the prevalence in midwestern US communities was high, 35%. The average household size was 4.31, and 100% of households had >1 child. Those findings are consistent with the continued increases of ESCR Enterobacterales in community settings, despite the successes of aggressive infection prevention and control campaigns in healthcare settings (*3*,*4*). We also found the presence of other major transmissible ARGs, such as fluoroquinolone, sulfonamide, fosfomycin, and tetracycline resistance genes, along with the presence of multiple conjugative plasmids among isolates (Table 3; Appendix Table 2). Previous studies that have investigated household transmission of MDR Enterobacterales were predominately among previously hospitalized adult patients with known colonization (*6*). 

Although ESBL-producing *K*. *pneumoniae* and *P*. *mirabilis* were recovered from households, we did not find a major presence of ESBL-producing *E. coli*. However, we did find the presence of high-risk *E. coli* clones (e.g., ST131, ST69, ST127, ST73) known for their epidemic potential and high potential for acquiring or having ARGs and MGEs.

Of note, the *Enterobacter cloacae* complex group of bacteria are ubiquitous in nature; however, these bacteria are most found in healthcare-associated infections in hospitalized patients or persons with antimicrobial or healthcare exposures. We were surprised to see such a high level of colonization in relatively healthy community households. In addition, most of our isolates within *Enterobacter cloacae* complex were *E. hormaechei*, which is often MDR and known to cause extraintestinal healthcare-associated infections, (e.g., urinary tract, bloodstream, and pneumonia), and can persist in healthcare environments (*44*). We found evidence of clustering of *E. hormaechei* within and between households, suggesting that household and community reservoirs might be a major source of community acquisition and spread of these pathogens.

The first limitation of our study is the relatively small sample size and large number of variables. We could not study the nonlinear and nonmonotonic effects of several count variables, and the suggested multivariate model might not be optimal for the given data. However, the relatively large prevalence of Enterobacterales has shown good statistical classification power of the study population. The multivariate model with 5 covariates showed good predictive power with 81.2% area under the curve. The model classified households with 75% sensitivity and 75% specificity when the threshold was 0.35. Second, because of the relatively small sample size of non-White households, we cannot provide details about the effect modification of race and suspect that race might represent a proxy for differences in socioeconomic status in the region of study. Although we show the link between pets, in particular dog ownership, and the diagnosis of ADHD and their association with household colonization with MDR Enterobacterales, many non-White households, predominantly Black, did not have pets or family members with ADHD diagnosis, limiting the ability for further analysis of those variables. Third, the parent study of the biorepository used for this analysis was initially designed to assess *S. aureus* household colonization, and households were selected on the basis of having a healthy child who had an *S. aureus* skin and soft tissue infection. Therefore, inguinal swab specimens were used to assess for household member colonization for MDR Enterobacterales because rectal or perirectal swab specimens were not collected in the parent study. Although ideally rectal or perirectal swab specimens would have been used, prior surveillance studies have demonstrated that the main reservoir for Enterobacterales is the gastrointestinal tract, and the inguinal folds are the most colonized skin site outside of the perirectal area because of the proximity to the rectum (*45*–*49*). A military study demonstrated that the inguinal folds were the most sensitive anatomic site for detecting MDR gram-negative colonization outside of the perirectum (negative predictive value 98%–100% for ESBL Enterobacterales) (*46*). The inguinal folds in young and diapered children also have a high burden of colonization and secondary infection with bacteria because of incontinence, excess moisture, and friction (*50*). Within our study population, most households (123 of 150) had children 0–5 years of age (*4*,*16*). In addition, our finding of a 4% inguinal fold colonization rate is consistent with prior US-based pediatric studies of intestinal colonization with ESCR (4.4%) and ESBL-producing (3.5%) Enterobacterales in healthy US children collected during well child clinic visits (*42*). Finally, whereas we can demonstrate clustering within households and communities, our retrospective analysis of a single time point cannot establish timing nor directionality of transmission within or between households, surfaces, and its members.

In conclusion, households might serve as a major contributor to the acquisition and spread of MDR Enterobacterales in the community. Factors associated with household colonization with MDR Enterobacterales include having a pet dog or children who attend daycare. Our current and future prospective One Health focused studies continue to investigate community reservoirs of MDR Enterobacterales in humans, animals, the household, and the natural environment. Our study emphasizes the necessity of investigating community reservoirs and spread of MDR Enterobacterales to learn more about how to mitigate potential sources associated with colonization and infection in the community. 

Appendix Additional information about role of households with children in community spread of multidrug-resistant Enterobacterales, St. Louis, Missouri, USA.
