# Supplementary material for: Role of Households with Children in Community Spread of Multidrug-Resistant Enterobacterales, St. Louis, Missouri, USA
Source: Emerg Infect Dis. 2026 Jun;32(6):914–24. doi: 10.3201/eid3206.251655 (PMC13245223; doi:10.3201/eid3206.251655)
Supplement: Appendix — Additional information about role of households with children in community spread of multidrug-resistant Enterobacterales, St. Louis, Missouri, USA. [file 25-1655-Techapp-s1.pdf]

Article DOI: https://doi.org/10.3201/eid3206.251655

EID cannot ensure accessibility for supplementary materials supplied by authors. Readers who have difficulty accessing supplementary content should contact the authors for assistance.

Role of Households with Children in the Community Spread of Multi-Drug Resistant Enterobacterales, St. Louis, Missouri, USA

Appendix

Appendix Table 1. Relatedness analysis for species and sequence types with more than 4 or more isolates

Species (ST)	Source of isolates (N)	SNV range
E. hormaechei (ST 50)	Environmental isolates (n=3) recovered from a kitchen faucet (n=1), an oven door handle (n=1) and a tablet (n=1) from the same household	2-3 SNVs
E. hormaechei (ST 108)	Environmental isolate (n=1) recovered from a kitchen faucet and an isolate from the index child bedsheet (n=1) from the same household	6 SNVs
K. pneumoniae (unknown ST)	Environmental isolates (n=2) recovered from an oven door handle (n=1) and from a tablet (n=1) from the same household	1 SNV
K. pneumoniae (ST 678)	Family member inguinal fold isolate (n=2) from the same family member	2 SNVs
S. marcescens (unknown ST)	Environmental isolates (n=4) isolated from a kitchen faucet (n=1) a microwave handle (n=1), a miscellaneous surface (n=1) and a phone (n=1) all from the same household	1-8 SNVs

ST: sequence type, SNV: single nucleotide variant

Appendix Table 2. *Enterobacter hormaechei* characteristics by isolate source

Characteristic	Total (N=47)	Environmental Source (N = 32)	Human Source (N = 15)
**MLST**			
Unknown	3 (6.4%)	2 (6.3%)	1 (6.7%)
102	1 (2.1%)	0 (0%)	1 (6.7%)
108	5 (11%)	3 (9.4%)	2 (13%)
113	1 (2.1%)	1 (3.1%)	0 (0%)
127	1 (2.1%)	1 (3.1%)	0 (0%)
1329	2 (4.3%)	1 (3.1%)	1 (6.7%)
133	1 (2.1%)	1 (3.1%)	0 (0%)
1344	1 (2.1%)	0 (0%)	1 (6.7%)
141	1 (2.1%)	1 (3.1%)	0 (0%)
190	2 (4.3%)	2 (6.3%)	0 (0%)
254	2 (4.3%)	2 (6.3%)	0 (0%)
267	1 (2.1%)	1 (3.1%)	0 (0%)
269	2 (4.3%)	2 (6.3%)	0 (0%)
286	2 (4.3%)	1 (3.1%)	1 (6.7%)
350	1 (2.1%)	1 (3.1%)	0 (0%)
421	1 (2.1%)	0 (0%)	1 (6.7%)
48	2 (4.3%)	2 (6.3%)	0 (0%)
50	5 (11%)	5 (16%)	0 (0%)
527	2 (4.3%)	0 (0%)	2 (13%)
604	2 (4.3%)	1 (3.1%)	1 (6.7%)
61	1 (2.1%)	1 (3.1%)	0 (0%)
68	4 (8.5%)	2 (6.3%)	2 (13%)
97	2 (4.3%)	1 (3.1%)	1 (6.7%)

Characteristic	Total (N=47)	Environmental Source (N = 32)	Human Source (N = 15)
974	2 (4.3%)	1 (3.1%)	1 (6.7%)
**Median AMR gene count per isolate [Q1, Q3]**	4 [3, 4]	4 [3, 4]	4 [3, 4]
**AMR resistance determinants**			
**Aminoglycoside AMR determinants**	3 (6.4%)	3 (2.4%)	0 (0%)
aadA2	1 (2.1%)	1 (0.8%)	0 (0%)
aph(3'')-Ib	1 (2.1%)	1 (0.8%)	0 (0%)
aph(6)-Id	1 (2.1%)	1 (0.8%)	0 (0%)
**B-lactam AMR determinants**	47 (100%)	32 (100%)	15 (100%)
blaACT (unspecified)	3 (6.4%)	1 (0.8%)	2 (13.0%)
blaACT-108	2 (4.3%)	0 (0%)	2 (13.0%)
blaACT-115	2 (4.3%)	1 (0.8%)	1 (6.7%)
blaACT-147	3 (6.4%)	2 (1.6%)	1 (6.7%)
blaACT-15	5 (10.6%)	5 (15.6%)	0 (0%)
blaACT-16	3 (6.4%)	2 (1.6%)	1 (6.7%)
blaACT-17	5 (10.6%)	4 (12.5%)	1 (6.7%)
blaACT-23	2 (4.3%)	1 (0.8%)	1 (6.7%)
blaACT-37	2 (4.3%)	2 (1.6%)	0 (0%)
blaACT-40	2 (4.3%)	2 (1.6%)	0 (0%)
blaACT-41	1 (2.1%)	1 (0.8%)	0 (0%)
blaACT-44	1 (2.1%)	1 (0.8%)	0 (0%)
blaACT-55	6 (12.8%)	3 (2.4%)	3 (20%)
blaACT-56	3 (6.4%)	2 (1.6%)	1 (6.7%)
blaACT-65	4 (8.5%)	4 (12.5%)	0 (0%)
blaACT-70	1 (2.1%)	0 (0%)	1 (6.7%)
**Fosfomycin AMR determinants**	31 (66.0%)	21 (65.6%)	10 (66.7%)
fosA	21 (44.7%)	21 (65.6%)	10 (66.7%)
**Quinolone AMR determinants**	47 (100%)	32 (100%)	15 (100%)
oqxB	47 (100%)	32 (100%)	15 (100%)
**Phenicol AMR determinants**			
catA	47 (100%)	32 (100%)	15 (100%)
**Sulfonamide AMR determinants**	2 (4.3%)	2 (1.6%)	0 (0%)
sul1	2 (4.3%)	2 (1.6%)	0 (0%)
**Tetracycline AMR determinants**	1 (2.1%)	1 (0.8%)	0 (0%)
tet(B)	1 (2.1%)	1 (0.8%)	0 (0%)
**Median plasmid count per isolate [Q1, Q3]**	1[1,3]	1[0,3]	2 [0,2.5]
**Plasmids**			
Col440I	10 (21%)	8 (47.1%)	2 (13%)
Col440II	1 (2.1%)	1 (3.1%)	0 (0%)
ColRNAI	2 (4.3%)	2 (6.3%)	
IncFIA(HI1)	5 (10.6%)	2 (6.3%)	3 (20%)
IncFIB(K)	3 (6.4%)	3 (4.7%)	0 (0%)
IncFIB(pB171)	1 (2.1%)	1 (3.1%)	0 (0%)
IncFIB(pECLA)	8 (17.0%)	5 (11%)	3 (20%)
IncFIB(pQil)	3 (6.4%)	1 (3.1%)	2 (13%)
IncFII(pECLA)	14 (29.8%)	8 (47.1%)	6 (40%)
IncFII(pKPX1)	1 (2.1%)	1 (3.1%)	0 (0%)
IncFII(Yp)	3 (6.4%)	2 (6.3%)	1 (4.5%)
IncHI2	2 (4.3%)	2 (6.3%)	0 (0%)
IncHI2A	2 (4.3%)	2 (6.3%)	0 (0%)
IncR	9 (19.1%)	4 (8.5%)	5 (33.3%)
IncX5	4 (8.5%)	4 (8.5%)	0 (0%)
repA	1 (2.1%)	1 (3.1%)	0 (0%)

Appendix Table 3. Descriptive statistics by households, by Race

Variable	Total HHs (n = 150)	African American and Other Races (40)	White (110)	Odds Ratio (95% CI)	p-value
HHs with resistant Enterobacterales	53 (35%)	23 (58%)	30 (27%)	0.28 (0.13, 0.58)	<0.01
Household (HH) size	4.31 (1.34)	4.68 (1.93)	4.18 (1.02)	0.77 (0.58, 1.00)	0.05
HH members' mean age (years)	20.86 (6.30)	18.72 (6.47)	21.64 (6.08)	1.09 (1.02, 1.18)	0.01
HH home size in square feet	1,785(1,021)	1,245 (991)	1,984 (962)	1 (1.00, 1.00)	<0.01
HH number of rooms	9.85 (3.35)	7.85 (2.63)	10.58 (3.30)	1.4 (1.21, 1.65)	<0.01
HH home square feet per person	441 (262)	303 (268)	492 (242)	1 (1.00, 1.01)	<0.01
HH size > 5					
No	130 (87%)	29 (73%)	101 (92%)		
Yes	20 (13%)	11 (28%)	9 (8.2%)	0.23 (0.09, 0.62)	0.00
HH homeownership					
No	56 (37%)	29 (73%)	27 (25%)		
Yes	94 (63%)	11 (28%)	83 (75%)	8.1 (3.67, 19.0)	<0.01
HHs with >= one Health condition					
No	24 (16%)	3 (7.5%)	21 (19%)		
Yes	126 (84%)	37 (93%)	89 (81%)	0.34 (0.08, 1.07)	0.10
HHs with >= one ADHD member					
No	94 (75%)	31 (84%)	63 (71%)		
Yes	32 (25%)	6 (16%)	26 (29%)	2.13 (0.84, 6.20)	0.13
HHs with >= one antibiotic prescription(12mo)					
No	85 (57%)	29 (73%)	56 (51%)		
Yes	65 (43%)	11 (28%)	54 (49%)	2.54 (1.18, 5.79)	0.02
HHs with >= one ER visit (12mo)					
No	10 (6.7%)	1 (2.5%)	9 (8.2%)		
Yes	140 (93%)	39 (98%)	101 (92%)	0.29 (0.02, 1.61)	0.24
HHs with >= one college degree					
No	86 (57%)	31 (78%)	55 (50%)		
Yes	64 (43%)	9 (23%)	55 (50%)	3.44 (1.55, 8.30)	0.00
HH with >= one professional degree					
No	102 (68%)	35 (88%)	67 (61%)		
Yes	48 (32%)	5 (13%)	43 (39%)	4.49 (1.76, 13.9)	0.00
HHs with >= one private insurance members					
No	35 (23%)	21 (53%)	14 (13%)		
Yes	115 (77%)	19 (48%)	96 (87%)	7.58 (3.33, 17.9)	<0.01
HHs with >= one Medicaid recipient					
No	104 (69%)	10 (25%)	94 (85%)		
Yes	46 (31%)	30 (75%)	16 (15%)	0.06 (0.02, 0.13)	<0.01
HHs with >= one minor					
No	38 (25%)	12 (30%)	26 (24%)		
Yes	112 (75%)	28 (70%)	84 (76%)	1.38 (0.60, 3.07)	0.43
HHs with >= one minor and attending daycare					
No	44 (29%)	10 (25%)	34 (31%)		
Yes	106 (71%)	30 (75%)	76 (69%)	0.75 (0.32, 1.66)	0.48
HHs with >= one Dog					
No	74 (49%)	30 (75%)	44 (40%)		
Yes	76 (51%)	10 (25%)	66 (60%)	4.5 (2.06, 10.6)	<0.01
HHs with >= one Cat					
No	125 (83%)	39 (98%)	86 (78%)		
Yes	25 (17%)	1 (2.5%)	24 (22%)	10.9 (2.18, 198)	0.02

Appendix Table 4. Odds ratios

Variable	Unadjusted Odds Ratio (95% CI)	Adjusted Odds Ratio (95% CI)	Absolute Change in OR
White Households	0.28 (0.13, 0.58)	0.18 (0.06, 0.49)	55.5%
Households with Private Insurance	0.35 (0.16, 0.77)	0.44 (0.16, 1.22)	20.5%
Households with one or more ADHD member	2.08 (0.91, 4.76)	3.47 (1.34, 9.41)	40.1%
Household with one or more minor attending daycare	1.25 (0.60, 2.69)	2.86 (1.07, 8.38)	56.3%
Households with one or more Dogs	1.45 (0.74, 2.86)	3.31 (1.27, 9.41)	56.2%

Note. Absolute change in OR = (adjusted OR – unadjusted OR) / adjusted OR.

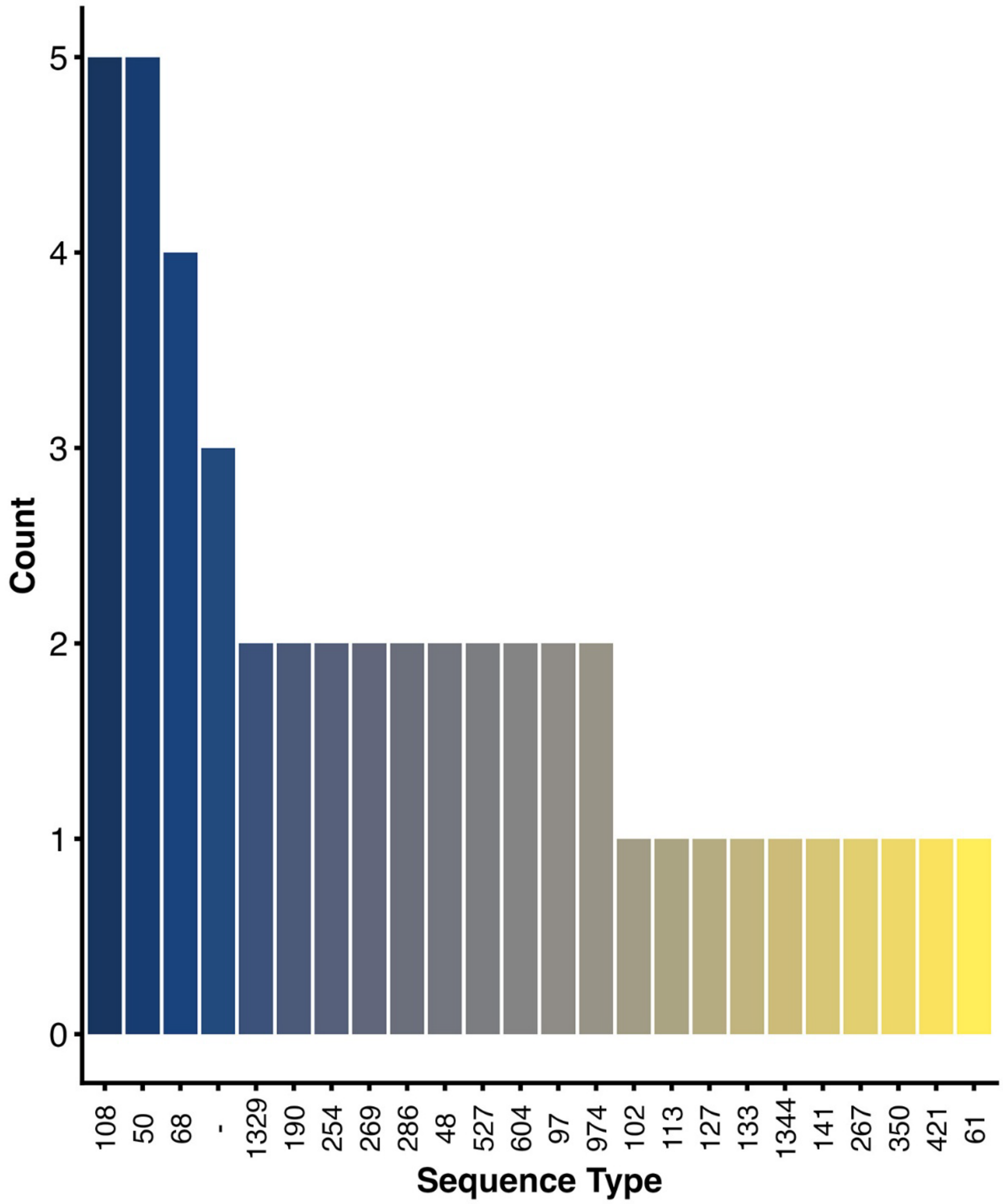

Figure. Case count by sequence type of multi-drug resistant Enterobacterales
